# Single-Molecule Real-Time and Illumina-Based RNA Sequencing Data Identified Vernalization-Responsive Candidate Genes in Faba Bean (*Vicia faba* L.)

**DOI:** 10.3389/fgene.2021.656137

**Published:** 2021-07-05

**Authors:** Xingxing Yuan, Qiong Wang, Bin Yan, Jiong Zhang, Chenchen Xue, Jingbin Chen, Yun Lin, Xiaoyan Zhang, Wenbiao Shen, Xin Chen

**Affiliations:** ^1^College of Life Sciences, Nanjing Agricultural University, Nanjing, China; ^2^Institute of Industrial Crops, Jiangsu Academy of Agricultural Sciences, Nanjing, China

**Keywords:** faba bean, transcriptome, SMRT sequencing, vernalization, *VfSOC1*

## Abstract

Faba bean (*Vicia faba* L.) is one of the most widely grown cool season legume crops in the world. Winter faba bean normally has a vernalization requirement, which promotes an earlier flowering and pod setting than unvernalized plants. However, the molecular mechanisms of vernalization in faba bean are largely unknown. Discovering vernalization-related candidate genes is of great importance for faba bean breeding. In this study, the whole transcriptome of faba bean buds was profiled by using next-generation sequencing (NGS) and single-molecule, real-time (SMRT) full-length transcriptome sequencing technology. A total of 29,203 high-quality non-redundant transcripts, 21,098 complete coding sequences (CDS), 1,045 long non-coding RNAs (lncRNAs), and 12,939 simple sequence repeats (SSRs) were identified. Furthermore, 4,044 differentially expressed genes (DEGs) were identified through pairwise comparisons. By Gene Ontology (GO) enrichment and Kyoto Encyclopedia of Genes and Genomes (KEGG) analysis, these differentially expressed transcripts were found to be enriched in binding and transcription factor activity, electron carrier activity, rhythmic process, and receptor activity. Finally, 50 putative vernalization-related genes that played important roles in the vernalization of faba bean were identified; we also found that the levels of vernalization-responsive transcripts showed significantly higher expression levels in cold-treated buds. The expression of *VfSOC1*, one of the candidate genes, was sensitive to vernalization. Ectopic expression of *VfSOC1* in *Arabidopsis* brought earlier flowering. In conclusion, the abundant vernalization-related transcripts identified in this study will provide a basis for future researches on the vernalization and faba bean breeding and established a reference full-length transcriptome for future studies on faba bean.

## Introduction

*Vicia faba* (L.), commonly known as faba bean or broad bean, is an annual or biennial herb of the family Fabaceae. It is a cross-pollinating diploid plant species (2*n* = 12) (Fuchs et al., 1998) that has a “giant genome” with an estimated genome size of 13 Gb (Johnston et al., 1999). The nuclear genome size of faba bean is one of the largest yet described among crop legumes, which is approximately 25 times greater than that of the model legume *Medicago truncatula* and 2.5 times greater than that of *Pisum sativum* (Rispail et al., 2010). Up to now, the genomic information of faba bean is blank, and there is no genome sequence for this species. Faba bean is one of the most effective nitrogen-fixing legumes for soil improvement abilities and is widely used for animal feed and human food, especially as a staple dietary protein source in North African and Middle Eastern cultures (O’Sullivan and Angra, 2016). Faba bean is also consumed as a vegetable crop. The immature faba bean is a high-quality vegetable, supplying well-balanced protein and carbohydrate together with numerous antioxidants and essential vitamins (Heather and Fawzy, 2010). China is the largest faba bean producer (in both sowing area and production) in the world, followed currently by Ethiopia, Australia, France, and Egypt (O’Sullivan and Angra, 2016). In the southern parts of China, faba bean is sown in areas between 21 and 35°N latitude from October to November and harvested from March to May (Ye et al., 2003). Winter faba bean comprised 85.5% of the total area and 78.2% of the total production of faba bean in China (Ye et al., 2003). It is the fourth most widely grown cool season legume after pea (*P. sativum*), chickpea (*Cicer arietinum*), and lentil (*Lens culinaris*) (Alharbi and Adhikari, 2020).

Vernalization plays an important role in the regulation of flowering time in higher plants for flower transformation, which is an evolutionarily adaptive mechanism preventing plant growth transition from the vegetative to the reproductive phase before winter and allowing flowering in favorable conditions in spring (Mahfoozi et al., 2006). The vernalization process has been thoroughly studied in *Arabidopsis* and in some winter crops such as wheat in the past years; however, the molecular regulatory mechanisms in other plants including faba bean are still largely unknown (Cao et al., 2017). In previous studies, researchers have identified some quantitative trait nucleotides (QTNs) or quantitative trait loci (QTLs) related to winter hardiness or days after flowering (DAF), but due to the limitation of genomic information, candidate genes were not found (Sallam et al., 2016; Catt et al., 2017).

In *Arabidopsis*, many reports indicated that flowering-time genes regulated vernalization. For example, FLOWERING LOCUS C (*FLC*) determines the extent of the vernalization response during flowering (Michaels and Amasino, 1999), FRIGIDA (*FRI*) activates *FLC* expression and drives *Arabidopsis* floral transition along the vernalization pathway (Sallam et al., 2016), VERNALIZATION1 (*VRN1*) encodes a protein that binds *FLC* in a non-sequence-specific manner and functions in the stable repression of *FLC* (Levy et al., 2002), and *VRN2* encodes a nuclear-localized zinc finger protein, maintains *FLC* repression, and serves as a mechanism for cellular memory (Gendall et al., 2001). Lately, *SOC1*, a MADS box transcription factor regulated by two antagonistic flowering regulators, CONSTANS (*CO*) and *FLC*, has acted as a floral activator and repressor (Lee and Lee, 2010). Recently, *TAF15b* (Eom et al., 2018), *PEP1* (Lazaro et al., 2018), *TRAF1a/1b* (Qi et al., 2017), and *VIN3* (Hepworth et al., 2018) were found to participate in vernalization directly or indirectly.

Due to the reading length, GC (guanine and cytosine) sensitivity, and other factors, the UniGene assembled by the traditional second-generation sequencing has incomplete transcripts. The third-generation sequencing—single-molecule, real-time (SMRT) sequencing—has good quality sequencing results, with an average reading length of 10–15 kb compared with the 1 kb sequenced by the second-generation sequencing technology, which can easily span the complete transcripts, and the full-length transcripts of messenger RNAs (mRNAs) can be obtained without assembly (Hepworth et al., 2018). In the present study, we used the SMRT sequencing technology to identify candidate vernalization genes and found that *VfSOC1* could promote flowering and response to vernalization. Furthermore, 50 putative flowering-related genes were identified and could be associated with vernalization. Thus, this study provides resources and directions for researchers to study the vernalization genes in faba bean.

## Materials and Methods

### Plant Materials and Treatment Conditions

Faba bean (*V. faba* L.) cv. Tongcanxian-6 seedlings were generated. Pacific Biosciences (PacBio, Menlo Park, CA, United States) isoform sequencing (Iso-Seq) libraries of faba bean were constructed using buds grown for 5 days to 1 cm (sample 0), buds and leaves with cold treatment (4°C) and dark culture for 14 days (sample 1), cold treated and dark cultured for 12 days then transferred to incubators (25°C/16 h and 20°C/8 h) for 2 days (sample 2), and cultured in incubators (25°C/16 h and 20°C/8 h) for 14 days (sample 3). For samples 1, 2, 3, and 0, and their T correspondence, the corresponding relations are as follows: T01–T03 represent three biological repetitions of sample 0; T04–T06 represent three biological repetitions of sample 1; T07–T09 represent three biological repetitions of sample 2; and T10–T12 represent three biological repetitions of sample 3. Finally 12 samples were sequenced. After treatment, the leaves were collected for RNA extraction. Faba bean total RNA was isolated using the Trizol reagent (Invitrogen, Foster City, CA, United States). Of the total RNA (equally mixed with RNAs from T01 to T12), 5 μg was reverse transcribed into complementary DAN (cDNA) using a SMARTer PCR cDNA synthesis kit (TaKaRa, Shiga, Japan), mRNA was purified from total RNA using poly-T oligo-attached magnetic beads, and was reverse transcribed into first-strand cDNA using a random hexamer primer. Then, DNA polymerase I and RNase H were used for second-strand cDNA synthesis. After end-blunting and adenylation of the 3′ ends, the DNA fragments were ligated with a sequencing adaptor and then size fractionation and selection (1–2, 2–3, and 3–6 kb) were performed using the BluePippin Size Selection System (Sage Science, Beverly, MA, United States). SMRTbell libraries were constructed with the PacBio DNA Template Prep Kit 2.0, and SMRT sequencing was then performed on the PacBio Sequel platform with three cells. Multiple size-fractionated cDNAs (1–2, 2–3, and 3–6 kb) were prepared to construct the faba bean Iso-Seq libraries.

### PacBio Long-Read Processing

Long reads from the PacBio platform were processed into error-corrected reads of insert (ROIs) using the ToFU pipeline^1^ with default parameters. ROIs were classified into non-full-length and full-length reads (including full-length non-chimeric and chimeric reads) based on the presence and location of the 3′ primer, 5′ primer, and polyA. We used ICE (iterative clustering for error correction^2^) to obtain consensus isoforms. Full-length consensus sequences from ICE were first polished using Quiver (Chin et al., 2013) and further corrected by Proovread (Hackl et al., 2014) software with the help of Illumina short reads. Then, the full-length transcripts with a post-correction accuracy above 99% were generated for further analysis. CD-HIT (Li and Godzik, 2006) was used to remove redundant high-quality full-length transcripts.

### SSR Detection

Simple sequence repeats (SSRs) of the transcriptome were identified using MISA software with default parameters^3^. The parameters were set as follows: one base repeat 10 times or more; two base repeats six times or more; three base repeats five times or more; four base repeats five times or more; five base repeats five times or more; and six base repeats five times or more. If the base repeats occur five or more times, they were regarded as microsatellite sequences. Moreover, if the distance between two microsatellites is less than 100 bp, the two microsatellites were treated as composite microsatellites.

### Coding Sequence Detection

TransDecoder^4^ was applied to identify candidate coding regions within the transcript sequences using the following criteria: (1) a minimum length of open reading frame (ORF) is found in a transcript sequence; (2) the log-likelihood score similar to what is computed by the GeneID^5^ software is > 0; (3) the above coding score is greatest when the ORF is scored in the first reading frame as compared to scores in the other five reading frames; (4) if a candidate ORF is found fully encapsulated by the coordinates of another candidate ORF, the longer one is reported. However, a single transcript can report multiple ORFs (allowing for operons, chimeras, etc.); and (5) optional, the putative peptide has a match to a Pfam (Protein family) domain above the noise cutoff score.

### Long Non-coding RNA Analysis

Four computational approaches—CPC (coding potential calculator) (Kong et al., 2007), CNCI (Coding-Non-Coding Index) (Sun et al., 2013), CPAT (Coding Potential Assessment Tool) (Wang et al., 2013), and the Pfam database (Finn et al., 2014)—were combined to sort the non-protein-coding RNA candidates from the putative protein-coding RNAs in the unknown transcripts. Putative protein-coding RNAs were filtered out using a minimum length and exon number threshold. Transcripts with lengths greater than 200 nt and have more than two exons were selected as long non-coding RNA (lncRNA) candidates and further screened using CPC/CNCI/CPAT/Pfam, which have the power to distinguish the protein-coding genes from the non-coding genes.

### Quantification of the Gene Expression Levels and Differential Expression Analysis

The clean reads of each RNA sequencing (RNA-Seq) library were aligned to the reference transcriptome to obtain unique mapped reads by using the Bowtie2^6^ software (Langmead and Salzberg, 2012), a comparison tool for sequencing reads aligned with long reference sequences and with output in the form of counts. The gene expression levels were estimated by as FPKM (fragments per kilobase of transcript per million fragments mapped) using RSEM (Li and Dewey, 2011) software. Mismatches of no more than two bases were allowed in the alignment. Clean data were first mapped back onto the assembled transcriptome, and then the read counts for each gene were obtained from the mapping results.

Differential expression analysis of two groups of samples was performed using the DESeq (Anders and Huber, 2012) R package. DESeq provides statistical routines for determining differential expression in digital gene expression data using a model based on a negative binomial distribution. The resulting *p*-values were adjusted using the Benjamini and Hochberg approach for controlling the false discovery rate (FDR). The critical standard for significant differentially expressed genes (DEGs) was set at FDR < 0.01 and fold change ≥ ≥ 2.

### Functional Annotation and Enrichment Analysis

Gene function was annotated based on the following databases: Nr (NCBI non-redundant protein sequences^7^), Pfam^8^, KOG/COG/eggNOG (Clusters of Orthologous Groups of Proteins)^9^, Swiss-Prot (a manually annotated and reviewed protein sequence database)^10^,^11^, KEGG (Kyoto Encyclopedia of Genes and Genomes)^12^, and GO (Gene Ontology)^13^.

GO enrichment analysis of the DEGs was implemented by the GOseq (Young et al., 2010) R package based on the Wallenius non-central hyper-geometric distribution, which can adjust for gene length bias in DEGs. We used KOBAS (Mao et al., 2015) software to test the statistical enrichment of the differential expression genes in the KEGG pathways.

### *Arabidopsis* Transformation and Growth Conditions

Faba bean total RNA was isolated using the Trizol reagent (Invitrogen, Foster City, CA, United States). The first-strand cDNA was then synthesized using M-MLV reverse transcriptase (Promega, Madison, WI, United States). PCR-amplified DNA fragments were linked to T Vector for sequences. Then, the full-length coding sequence of *VfSOC1* was cloned into pEarley Gate103 driven by the 35S promoter. The plasmid was transformed into the *Agrobacterium tumefaciens* EHA105 strain. Overexpressed transgenic plants were generated by the floral dipping method (Qi et al., 2017) in the *Col* background through *A. tumefaciens*-mediated transformation and subsequent selection by hygromycin on 1/2 Murashige and Skoog medium.

Wild-type *Arabidopsis thaliana* (*Col-0*) was used as the background, and transgenic lines were grown on soil under long days (16-h light/8-h dark) at 24 ± 2°C.

### Expression Analysis

For the semi-quantitative RT-PCR analysis of *VfSOC1* expression in transgenic *Arabidopsis* lines, *VfSOC1* was detected by the primers *VfSOC1*-F-1 (GACCGATACCGCAGTCATAGCCG) and *VfSOC1*-R-1 (GCATGACATTT TCAGCAACTAGGGCT). *Actin2* (*At3g18780*) was used as the reference gene (Actin2-F: TCCTTTGTTGCTGTTGACTACG, Actin2-R: ATTTTCT GTGAACGATTCCT GG).

## Results

### Long-Length Transcriptome of Faba Bean

**FIGURE 1:**
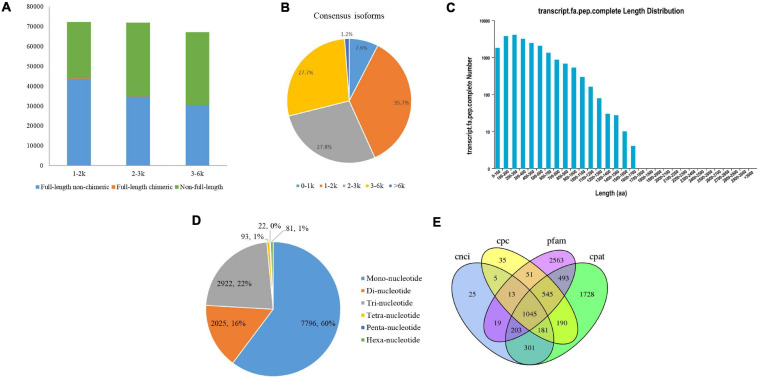
PacBio Iso-Seq Full-length transcriptome analysis. **(A)** Distributions of full length non-chimera (FLNC), full length chimera and non-full length transcripts in faba bean bud libraries. **(B)** Length distributions of consensus isoforms. **(C)** Length distribution of CDS. **(D)** Type distribution of SSRs. **(E)** Venn diagram showed the number of lncRNA identified by four approaches.

A total of 8.62 Gb of clean data were obtained from all three cells with 450,876 raw polymerase reads. After filtering the adaptor and low-quality sequences, a total of 3,671,393 subreads were obtained (Supplementary Table 1). A total of 233,493 high-quality ROIs were further generated from circular consensus sequencing (CCS) after filtering with full passes and accuracy. The numbers of ROIs from the faba bean libraries were 82,216 for 1–2 kb, 80,070 for 2–3 kb, and 71,207 for 3–6 kb. In total, 109,128 full-length non-chimeric (FLNC) reads were produced from the ROIs of the faba bean libraries, with average lengths of 1,346, 2,363, and 3,283 bp in the corresponding faba bean libraries (Figure 1A and Supplementary Table 2).

After classification and correction by the ICE and Quiver programs, 40,046 high-quality (accuracy > 0.99) and 10,727 low-quality polished consensus isoforms were generated from the ROIs. Consensus isoforms were further corrected using Proovread (Hackl et al., 2014) software with the help of Illumina short reads. Ultimately, a total of 29,203 unigene transcripts were obtained after CD-HIT classification. The length distribution of the polished consensus isoforms is shown in Figure 1B, with almost 91.2% of the sequences ranging from 1 to 6 kb.

### Faba Bean Transcriptome of NGS Short Reads

We used the same treated samples for SMRT, and the Illumina RNA-Seq libraries constructed from faba bean buds with different treatments were sequenced to correct the polished isoforms and to quantify the full-length transcripts obtained from PacBio Iso-Seq. After the trimming process, 86.99 Gb clean data were obtained from all samples. Over 214 million short reads were successfully mapped back to the full-length transcripts of PacBio Iso-Seq with an average mapping ratio of 73.3% (Supplementary Table 3), which suggested that the full-length transcripts derived by the PacBio Iso-Seq method represented most of the genetic information of faba bean.

### Functional Annotations

Databases such as Nr, Swiss-Prot, GO, COG, KOG, Pfam, and KEGG were used to perform functional annotations on the 29,203 unigenes. A total of 27,903 unigenes (95.5%) were successfully matched to known sequences in at least one database (Supplementary Table 4). There were 95.4% unigenes matched in the Nr database, 76.9% in Swiss-Prot, and 41.9% in COG.

A total of 12,763 (43.7%) unigenes were annotated with 125 pathways in the KEGG pathway analysis. GO terms were assigned to 18,040 unigenes (61.8%) with 19 biological processes, 17 cellular components, and 14 molecular functions. In the biological processes, the top three GO terms were “metabolic process,” “cellular process,” and “single-organism process.” In the class of cellular component, “cell part” was dominant, and then “cell” and “organelle.” High percentages of the unigenes were enriched in “catalytic activity,” “binding,” and “transporter activity” in the class of molecular functions (Supplementary Table 5).

### CDS, SSR, and lncRNA Prediction

The candidate coding sequence (CDS) in the PacBio Iso-Seq transcripts was analyzed by retaining a minimum length of ORFs using the TransDecoder software. As shown in Figure 1C, a total of 21,098 complete CDS were obtained from faba bean transcripts with lengths ranging from 50 to 1,669 bp and an average length of 390 bp.

SSRs are short (1–6 bp) tandem repeat DNA sequences. They are also known as microsatellites. In this study, we identified 12,939 SSRs in 8,939 unigenes (30.6% of the total unigenes), including six types of SSRs: mononucleotide (7,796, 60.3% of all SSRs), dinucleotide (2,025, 15.7%), trinucleotide (2,922, 22.6%), tetranucleotide (93, 0.7%), pentanucleotide (22, 0.2%), and hexanucleotide (81, 0.6%) (Figure 1D). Among them, 1,373 SSRs present in compound formation.

Four computational approaches—CPC, CNCI, CPAT, and Pfam—were combined to sort the non-protein-coding RNA candidates from the putative protein-coding RNAs in the faba bean transcripts and then identify the lncRNAs. A total of 1,045 lncRNA transcripts were predicted by these four methods (Figure 1E). The lengths ranged from 541 to 14,958 bp, with an average length of 1,860 bp.

### Identification of Differentially Expressed Genes

**FIGURE 2:**
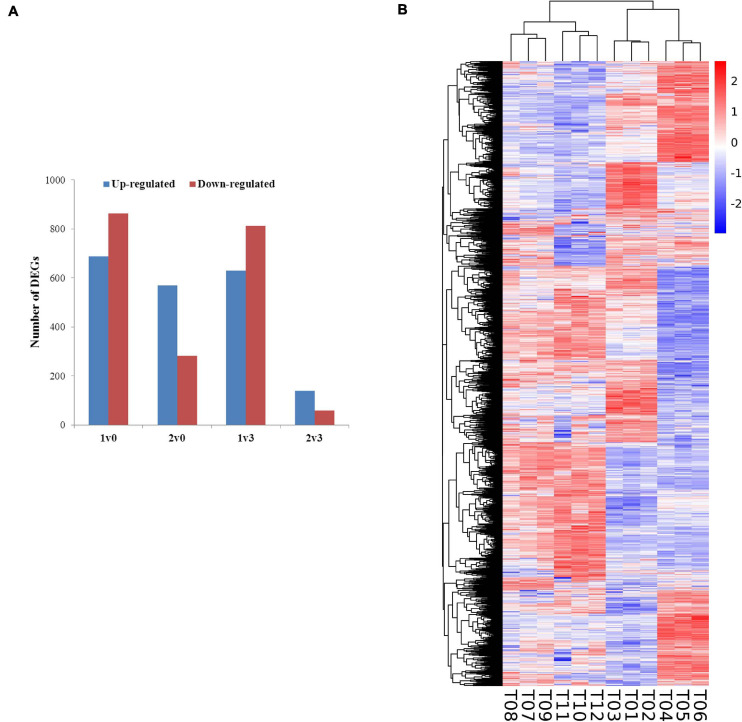
Gene expression profiles of faba bean buds. **(A)** Numbers of DEGs in pairwise comparisons of the four treatments. **(B)** Heatmap diagrams showed the relative expression levels of DEGs among the four treatments.

The expression levels of the transcripts from the PacBio Iso-Seq libraries of four groups of faba bean buds with different treatments were quantified using the FPKM method. Differences in expression were examined to identify the transcripts that may participate in the vernalization of faba bean. A total of 4,044 DEGs (fold change > 2, FDR < 0.01) in faba bean were identified through pairwise comparisons (Figure 2A), of which 1,553, 851, 1,443, and 197 DEGs were found between buds with cold treatment and dark culture (sample 1) and 1 cm control (sample 0), cold treated then transferred to incubators (sample 2) and 1 cm control (sample 0), cold treated and dark cultured (sample 1) and those cultured in incubators (sample 3), and cold treated then transferred to incubators (sample 2) and those cultured in incubators (sample 3), respectively. Six hundred eighty-nine genes were upregulated and 864 genes were downregulated under sample 1 compared with sample 0. Among these genes, the expression levels of *VfADO3* (log_2_FC = 3.32, FDR = 6.56e−08), *VfCRY1* (log_2_FC = 1.87, FDR = 3.17e−04), *VfSOC1* (log_2_FC = 3.97, FDR = 0.003), and *VfGI* (log_2_FC = 5.05, FDR = 5.94e−06) were significantly higher in sample 1 than those in sample 0 (Supplementary Table 7). A comparison of sample 2 with sample 0 revealed 569 genes that were upregulated (Supplementary Table 9), including *VfCDF3* (log_2_FC = 2.69, FDR = 9.79e−04), and 282 genes that were downregulated, including *VfADO3* (log_2_FC = −3.54, FDR = 1.83e−04) and *VfCOL9* (log_2_FC = −3.63, FDR = 1.19e−07) (Supplementary Table 7). Six hundred thirty genes were upregulated, including *VfCDF3* (log_2_FC = 2.31, FDR = 0.009), and 813 genes were downregulated, including *VfADO3* (log_2_FC = −6.77, FDR = 7.41e−18), *VfSOC1* (log_2_FC = −5.55, FDR = 1.15e−04), *VfPHYA* (log_2_FC = −7.30, FDR = 2.14e−05), and *VfGI* (log_2_FC = −3.54, FDR = 1.83e−04), under sample 1 compared with sample 3 (Supplementary Table 6). Moreover, 139 genes were upregulated and 58 genes were downregulated under sample 2 compared with sample 3 (Supplementary Table 8); however, only *VfPHYA*, the flowering-related gene, was significantly downregulated (log_2_FC = −4.99, FDR = 6.24e−05). Lastly, we chose *VfSOC1* for further functional analysis.

Furthermore, a general overview of the expression patterns was visualized in a heat map (Figure 2B), which provided an overall understanding of the changes in gene expression.

### Functional Classification of the DEGs by GO and KEGG Pathway Analyses

**FIGURE 3:**
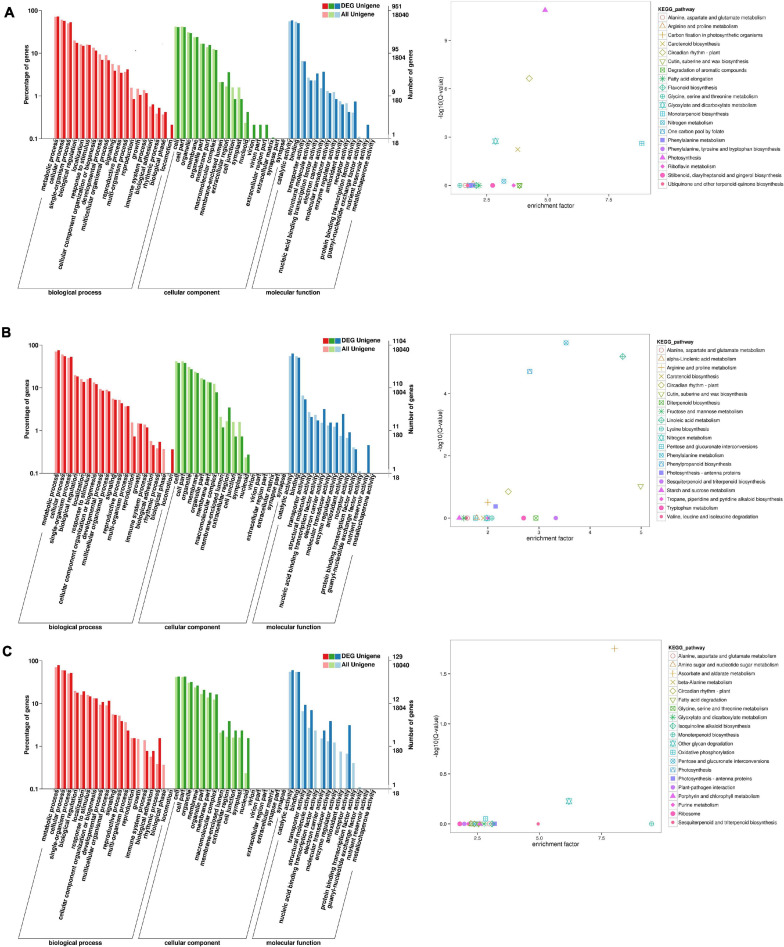
GO and KEGG enrichment analyses of DEGs (move to Appendix). **(A)** GO and KEGG enrichment analyses of DEGs between cold treated dark cultured and incubators cultured faba bean buds. **(B)** GO and KEGG enrichment analyses of DEGs between cold treated dark cultured faba bean buds and control. **(C)** GO and KEGG enrichment analyses of DEGs between cold treated dark cultured then transferred to incubators and incubators cultured faba bean buds.

To identify the flowering-related genes in faba bean, the DEGs were examined with GO and KEGG pathway analyses. The three faba bean bud samples with cold treatment and dark culture were compared to the three matched samples of faba bean bud cultured in incubators. Of the DEGs, 1,443 were screened out, with log2 fold changes ranging from −9.9 to 12.1 (Supplementary Table 6). The DEGs in comparison were enriched in three main GO categories. In the biological process category, the GO terms were significantly enriched in reproduction and rhythmic process. In the molecular function category, binding transcription factor activity, electron carrier activity, receptor activity, and nutrient reservoir activity were significantly enriched. These proteins may play critical roles as transcription factors or at the transcriptional level in low-temperature stress and flowering signaling pathways (Figure 3). As for the KEGG pathway analysis, “photosynthesis,” “circadian rhythm—plant,” “monoterpenoid biosynthesis,” “glyoxylate and dicarboxylate metabolism,” “carotenoid biosynthesis,” and “nitrogen metabolism” were significantly enriched. Changes of the flowering phenotype of faba bean may be associated with circadian rhythm (Figure 3). In addition, 58 unigenes were specifically highly expressed in faba bean buds with cold treatment and dark culture, while no expression was observed in any of the matched incubator-cultured samples. In contrast, 22 unigenes were highly expressed in the three incubator-cultured samples exclusively (Figure 3A and Supplementary Table 6).

A total of 1,553 significant DEGs (Figure 3B and Supplementary Table 7) were identified in faba bean between cold-treated and dark-cultured faba bean buds and 1 cm control, with log2 fold changes ranging from −9.0 to 10.3. The GO terms were clustered. In the biological process category, the significantly enriched GO terms were similar to those enriched in cold-treated dark-cultured buds compared to buds cultured in incubators. As for molecular function, DEGs were specifically involved in “antioxidant activity” (Figure 3B). Enrichment analysis based on the KEGG database revealed that the most enriched terms were “phenylalanine metabolism,” “phenylpropanoid biosynthesis,” and “cutin, suberine, and wax biosynthesis.” In addition, 22 and 18 unigenes were exclusively highly expressed in the cold-treated dark-cultured and control faba bean buds (Supplementary Table 7), respectively.

Three faba bean bud samples from the cold treatment and dark culture then transferred to incubators group were compared to the three matched samples cultured in incubators in order to screen the key genes regulating the growth of faba bean that maintained expression. A total of 197 DEGs (Figure 3C and Supplementary Table 8) were identified with log2 fold changes ranging from −11.7 to 10.1. The most significantly enriched GO terms in biological process were “rhythmic process” and “growth.” In the molecular function category, the DEGs were significantly enriched in “structural molecule activity,” “molecular transducer activity,” “enzyme regulator activity,” and “receptor activity.” In addition, 21 unigenes were highly expressed in buds that were cold treated and dark cultured then transferred to incubators specifically, but were not expressed in the other treatments; seven unigenes were exclusively expressed in faba bean buds cultured in incubators.

### Identification and Expression Analysis of Flower-Related DEGs in Faba Bean

**FIGURE 4:**
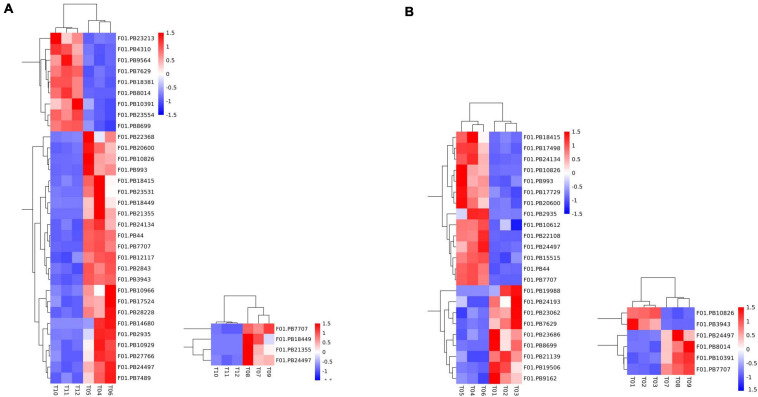
Expression pattern analyses of the flowering-related DEGs. Heatmap of the expression patterns of 50 flowering-related DEGs in pairwise comparisons. **(A)** 32 flower-related genes differentially expressed between cold treated dark cultured and incubators cultured faba bean buds. **(B)** 23 genes were significantly highly expressed with cold treatment and dark culture.

A total of 50 DEGs were functionally annotated as putative flowering-related genes (Supplementary Table 10). We analyzed the expression patterns of these differentially expressed transcripts (Figure 4) and identified 32 flower-related genes differentially expressed between faba bean buds that were cold treated dark cultured and buds cultured in incubators. Among them (Figure 4A), 23 genes were significantly highly expressed with cold treatment and dark culture (Figure 4B). *SOC1* (suppressor of overexpression of *CO*) is a key gene involved in the flowering pathway, while ADO3 (Adagio protein 3) forms a complex with “GIGANTEA” (GI) to regulate “*CONSTANS*” (*CO*) expression and promotes *CO* expression during the light period of long daylight by decreasing the stability of *CDF1* and *CDF2* and by interacting directly with the CO protein and stabilizing it (Imaizumi et al., 2005). These genes may affect flower development when the faba bean buds were treated with cold.

In cold-treated and dark-cultured buds vs. the 1 cm control, 23 flower-related genes were identified, 14 of which were significantly highly expressed in cold-treated faba bean. These three genes significantly highly expressed in cold-treated vs. buds cultured in incubators—*SOC1*, *ADO3*, and *GI*—were also highly expressed (Figure 4B). Interestingly, *COL5* (zinc finger protein CONSTANS-LIKE 5) and *CRY1* (cryptochromes) were also upregulated, and the stability of CO is positively regulated by the blue light receptor cryptochrome CRY1, suggesting that these genes may be involved in the process of flowering.

Only four flowering-related DEGs were identified in the samples treated with cold and dark cultured then transferred to incubators compared with the incubator-cultured samples, and all of them were upregulated in the former, including *PHYA* (*Phytochromes A*), which could positively regulate the stability of the CO protein (Figure 4A). *PHYA* has the same function as *CRY1*. Together, these two genes may be involved in the flowering pathway.

### Validation of Flowering-Related Genes

**FIGURE 5:**
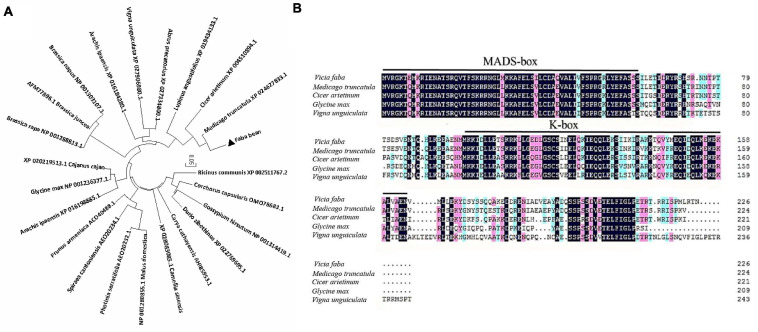
Phylogenetic analysis of *VfSOC1* and amino acid sequence alignments with closely related proteins. **(A)** Neighbor-joining analysis of SOC1 proteins, *Numbers* indicate the percentage of consensus support. **(B)** Amino acid alignments of VfSOC1 and ortholog Proteins from *Medicago truncatula*, *Cicer arietimum*, *Glycine max, and Vigna unguiculata*. Conserved residues are shown in *black* and partial conservation is shown in *pink* and *light blue*. The MADS and K-box domains are indicated with *black lines*, respectively.

**FIGURE 6:**
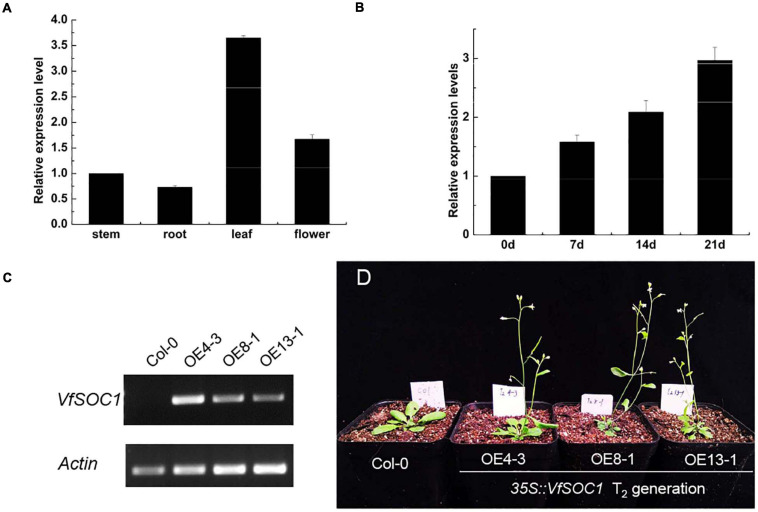
Functional analysis of *VfSOC1*. **(A)** Expression pattern of VfSOC1 in stem, root, leaf and flower in faba bean. **(B)** Time-course of expression of *VfSOC1* in leaf after vernalization, **(C)** Semi-quantitative expression analysis *VfSOC1* in three independent overexpression lines. The *Arabidopsis* Actin gene was amplified as a control. **(D)** Overexpression of *VfSOC1* in wild-type (Col-0) promote flowering. All plants are planted under long days.

The ORF of *VfSOC1* had 678 bases that encoded 226 amino acids. The full-length *VfSOC1* amino acid sequence was used as a query to search for predicted orthologs in GenBank. Phylogenetic analysis showed that *VfSOC1* is most similar to *MtSOC1c* in *Medicago*, with 86.5% identity (Figure 5A). *VfSOC1* contained a highly conservative N-terminal MADS domain and a *Keratin-like* domain (Figure 5B). In *Medicago*, the *SOC1* family genes had distinct and specific expression patterns (REF) (Jaudal et al., 2018). Thus, we determined the expression of *VfSOC1* in different tissues by RT-PCR; the results showed that *VfSOC1* was constitutively expressed in both vegetative and reproductive organs with high abundance in the leaves and flowers (Figure 6A). Since flowering is promoted by vernalization in faba bean, we checked the expression level of *VfSOC1* in the leaf under continuous low-temperature treatment. A pronounced elevation of the *VfSOC1* transcript was observed at day 7, and the expression level showed a persistent increase (Figure 6B). This indicated that *VfSOC1* expression is sensitive to vernalization in faba bean. To investigate the function of *VfSOC1*, a series of *Arabidopsis* transgenic plant lines expressing the CDS of *VfSOC1* driven by the CaMV 35S promoter were generated in the wild-type (*Col-0*) background. Three individual homozygous lines were obtained at T_2_ generation, with an increasing expression level of *VfSOC1* (Figures 6C,D). Compared with the wild type, all three lines (*OE4-3*, *OE8-1*, and *OE13-1*) displayed an earlier flowering under the long-day condition (Figure 6D).

In summary, these results corroborate that *VfSOC1* can promote flowering and may play an important role in response to the vernalization of faba bean.

## Discussion

Compared with the next-generation sequencing (NGS) platform, the third-generation sequencing platform, SMRT sequencing, developed by PacBio RS is more appropriate for long reads with an average read length of greater than 10 kb, and the real length can reach 60 kb^14^. After the correction procedure (self-corrected *via* CCS reads and corrected with the help of NGS reads), the error rate of SMRT sequencing is expected to be 1%. Furthermore, the assembly of the short NGS reads often encounters the complication of lacking reference genome sequences. This problem seems more severe for faba bean, which has a “giant genome” with an estimated genome size of greater than 13 Gb (2*n* = 12). Also, repetitive sequences in faba bean, such as retrotransposons and microsatellites, bring in further challenges for the accuracy of the short-read assembly. PacBio Iso-Seq can obtain a collection of high-quality full-length transcripts as reference sequences without assembly, thus overcoming these difficulties (Li and Godzik, 2006; Chin et al., 2013; Hackl et al., 2014).

In this study, NGS and the SMRT sequencing technology were combined to generate full-length transcriptomes of faba bean, and differentially expressed full-length transcripts of faba bean were identified and characterized. A total of 29,203 full-length faba bean transcripts were identified with an average length of 2,401.89 bp. Among these unigenes, 27,903 were functionally annotated with a functional annotation ratio of 95.5%. The high functional annotation ratio suggested that Iso-Seq could provide higher accuracy and is more effective in recovering full-length transcripts.

Vernalization plays an important role in the regulation of flowering time in higher plants, which is an evolutionarily adaptive mechanism preventing plant growth transition from the vegetative to the reproductive phase before winter and allowing flowering in favorable conditions in spring (Zhao et al., 2006). All winter crops generally have a vernalization requirement. In the past years, the vernalization process has been thoroughly studied in *Arabidopsis* (Michaels and Amasino, 1999; Gendall et al., 2001; Levy et al., 2002; Lee and Lee, 2010; Fadina and Khavkin, 2014; Eom et al., 2018) and in winter cereals such as wheat (Yan et al., 2003, 2006; Dubcovsky et al., 2006). However, the molecular regulatory mechanisms of vernalization in faba bean are still largely unknown. Recent advances in sequencing technologies enable the generation of large volumes of sequencing data efficiently and cost effectively. Transcriptome analysis is a rapid approach to obtaining expressed gene sequences for faba bean, which has a large and poorly characterized genome. In this study, the transcriptome of faba bean under vernalization or chilling treatment was sequenced using the combined approach of short-read NGS and long-read SMRT technology, and the genes were functionally annotated. It provided a basis for future researches on the vernalization responses and breeding of faba bean and established a reference full-length transcriptome for future studies on faba bean.

As SSR markers are important in variety identification, in this study, we identified 12,939 SSRs, although these SSRs have not been verified in natural populations. The SSRs were identified in vernalization-related traits, which would be useful in winter hardiness, DAF and yield traits in faba bean. In addition, the SSR markers identified in this study are useful for evaluating genetic relationships and the population structure of winter ecotype faba bean accessions of different origins and will facilitate utilizing various genetic resources.

**FIGURE 7:**
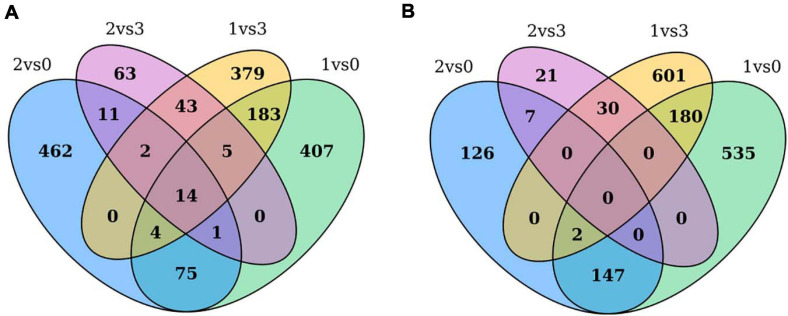
Gene expression profile between pairwise comparisons. Venn diagram showed the number of DEGs in different treatments. **(A)** Up-regulated DEGs between cold treated and incubators cultured were also among the genes differentially expressed between cold treated and 1 cm control. **(B)** Down-regulated DEGs between cold treated and incubators cultured were also among the genes differentially expressed between cold treated and 1 cm control.

Through pairwise comparisons, a total of 29,203 DEGs were identified in this study. To reveal the underlying molecular mechanism of buds of faba bean to flower under cold treatment, GO and KEGG pathway analyses of DEGs were conducted. DEGs were enriched in pathways of “binding transcription factor activity,” “electron carrier activity,” “receptor activity,” and “nutrient reservoir activity.” Among the 29,203 DEGs identified, 206 of the 630 upregulated DEGs between those cold treated and those cultured in incubators were also among the genes differentially expressed between those cold treated and the 1 cm control (Figure 7A). In addition, 182 of the 813 downregulated DEGs between those cold treated and incubator cultured were also among the genes differentially expressed between those cold treated and the 1-cm control (Figure 7B). Among these genes, the expression levels of *VfADO3*, *VfCRY1*, *VfSOC1*, and *VfGI* were significantly higher in sample 1 than those in sample 0 (Supplementary Table 7). Moreover, *VfSOC1* (log_2_FC = −5.55, FDR = 1.15e−04) was downregulated under sample 1 compared with sample 3 (Supplementary Table 6). In *Arabidopsis*, *SOC1* could be regulated by two antagonistic flowering regulators, CONSTANS (*CO*) and *FLC*, and acts as a floral activator and repressor (Lee and Lee, 2010). In order to study the function of *VfSOC1*, we implemented the transgenic work.

In the transgenic *Arabidopsis* lines (*OE4-3*, *OE8-1*, and *OE13-1*), all three lines displayed earlier flowering compared with *Col-0* (Figures 6D,E), which indicated that *VfSOC1* could promote flowering. Through this study, candidate genes related to vernalization were identified (Sallam et al., 2016; Catt et al., 2017).

Moreover, the molecular mechanism of *VfSOC1* is worth studying in the future. Further physiological and molecular experiments should be conducted to provide more precise insights into the mechanisms of vernalization in faba bean.

## Data Availability Statement

The datasets presented in this study can be found in online repositories. The names of the repository/repositories and accession number(s) can be found below: NCBI BioProject, accession no: PRJNA704197.

## Author Contributions

XY conceived the project. WS and XC supervised the work. XY, BY, and JZ performed most of the experiments, with assistance from QW, CX, JC, YL, and XZ. XC, XY, and QW wrote the manuscript, with contributions from all authors. All authors analyzed the data.

## Conflict of Interest

The authors declare that the research was conducted in the absence of any commercial or financial relationships that could be construed as a potential conflict of interest.

## Footnotes

1 http://github.com/PacifcBiosciences/cDNA_primer/

2 https://www.pacb.com/productsand-services/analytical-sofware

3 http://pgrc.ipk-gatersleben.de/misa/

4 https://github.com/TransDecoder/TransDecoder/releases

5 http://genome.crg.es/sofware/geneid/

6 http://bowtie-bio.sourceforge.net/bowtie2/index.shtml

7 http://www.ncbi.nlm.nih.gov/RefSeq/

8 http://pfam.sanger.ac.uk/

9 http://www.ncbi.nlm.nih.gov/COG/

10 http://www.expasy.ch/sprot/

11 http://www.ebi.ac.uk/swissprot/Boeckmann

12 http://www.genome.jp/kegg/

13 http://www.geneontology.org

14 www.pacb.com/smrt-science/smrt-sequencing/read-lengths/

## Supplementary Material

Supplementary Table 1 PacBio Iso-Seq data statistics.

Supplementary Table 2 Summary of reads of insert (ROIs).

Supplementary Table 3 Mapping statistics of Illumina RNA-seq data.

Supplementary Table 4 Statistics of annotated transcripts in different databases.

Supplementary Table 5 GO enrichment statistics.

Supplementary Table 6 DEGs expressed in cold treated and dark cultured (sample 1) compared with incubators cultured faba bean buds (sample 3).

Supplementary Table 7 DEGs expressed in cold treated and dark cultured faba bean buds (sample 1) compared with control (sample 0).

Supplementary Table 8 DEGs expressed in cold treated and dark cultured then transferred to incubators (sample 2) compared with incubators cultured faba bean buds (sample 3).

Supplementary Table 9 DEGs expressed in cold treated then transferred to incubators (sample 2) compared with control (sample 0).

Supplementary Table 10 Flowering-related DEGs identified in this study.
